# P04.09. Acupuncture and chiropractic utilization among chronic musculoskeletal pain patients at a health maintenance organization

**DOI:** 10.1186/1472-6882-12-S1-P279

**Published:** 2012-06-12

**Authors:** C Elder, L DeBar, C Ritenbaugh, M Aickin, R Deyo, R Meenan, J Dickerson, J Webster, B Yarborough

**Affiliations:** 1 Kaiser Permanente Center for Health Research, Portland, USA; 2 University of Arizona, Tucson, USA; 3 Oregon Health and Science University, Portland, USA

## Purpose

To describe acupuncture and chiropractic (acu/chiro) use among 11,960 chronic musculoskeletal pain patients enrolled in a Pacific Northwest Health Maintenance Organization (HMO).

## Methods

We identified 119,732 HMO members with chronic musculoskeletal pain from electronic medical records data. Patients were contacted by mail and invited to complete an online survey. Those not responding were either contacted by email or mailed a paper copy of the survey. Survey questions included items related to diagnosis, acu/chiro utilization and payment, and communication about acu/chiro use with HMO clinicians.

## Results

Of 119,732 patients invited to participate, 11,960 completed the survey. Self reported pain diagnoses included back pain (65%), joint pain (56%), arthritis (53%), extremity pain (53%), neck pain (37%), muscle pain (30%), headaches (21%), fibromyalgia (13%), and abdominal or pelvic pain (10%). Participants were predominantly caucasian (90%) and female (58%), with mean age of 59 years. Roughly a quarter of participants (n=3,169) reported acupuncture use, while slightly more participants (n=4,712) reported chiropractic use. Of those using acupuncture, 39% did not discuss acupuncture use with their HMO clinician, and 82% paid out-of pocket for at least a portion of the care. Of those using chiropractic services, 38% did not discuss chiropractic use with their HMO clinician, and 91% paid out-of-pocket for at least a portion of the care. The most common reason given for out-of-pocket payment to acu/chiro providers was that insurance coverage didn’t cover such care or required a co-payment. Patients with a self referral acu/chiro benefit were more likely to use acu/chiro than those without such coverage (p<.0001), but were not more likely to communicate acu/chiro use to HMO clinicians (p=.13).

## Conclusion

Acu/chiro use is prevalent among HMO patients with chronic musculoskeletal pain. However, such use frequently is not reported to HMO clinicians, even by patients having a self referral acu/chiro benefit.

